# Topical Application of Hangeshashinto (TJ-14) in the Treatment of Chemotherapy-Induced Oral Mucositis

**DOI:** 10.4021/wjon263w

**Published:** 2011-01-01

**Authors:** Toru Kono, Machiko Satomi, Naoyuki Chisato, Yoshiaki Ebisawa, Manabu Suno, Toshiyuki Asama, Hidenori Karasaki, Kazuo Matsubara, Hiroyuki Furukawa

**Affiliations:** a Division of Gastroenterologic and General Surgery, Department of Surgery, Asahikawa Medical University, Asahikawa, Japan; b Division of Chemotherapy, Higashi-Asahikawa Hospital, Asahikawa, Japan; c Department of Hospital Pharmacy and Pharmacology, Asahikawa Medical University, Asahikawa 078-8510, Japan

**Keywords:** Oral mucositis, Chemotherapy, Hangeshashinto, TJ-14, Topical treatment

## Abstract

**Background:**

The optimal treatment of chemotherapy-induced oral mucositis is not well established. A recent study showed that hangeshashinto (TJ-14) might be useful for periodontal disease via downregulating pro-inflammatory prostaglandins in the cyclooxygenase pathway in human. Our study aimed to determine whether TJ-14 is effective in the management of chemotherapy-induced oral mucositis.

**Methods:**

Fourteen patients afflicted with chemotherapy-induced oral mucositis during mFOLFOX6 or FOLFIRI treatment for metastasis of advanced colorectal cancer were randomly assigned to topical TJ-14 treatment thrice daily for 7 days. Patients prepared a 50 ml solution with 2.5 g of TJ-14 dissolved in tap water and rinsed their oral mucosa for more than 5 seconds and then expectorated it. TJ-14 was also topically applied with a cotton pellet on the mucosal lesions. The severity of oral mucositis was evaluated using the Common Terminology Criteria for Adverse Events version 4 before and after one-week TJ-14 treatment.

**Results:**

After the one-week topical treatment with TJ-14, thirteen of the fourteen patients (92.8 %) showed improvements in oral mucositis, with significantly decreased mean CTCAE grades (*P* = 0.0012). Compared to baseline, none of the patients’ CTCAE grades worsened. The compliance of TJ-14-treatment was good and side effects from TJ-14 were not observed.

**Conclusions:**

Topical application of TJ-14 may have therapeutic effects in patients with chemotherapy-induced oral mucositis via downregulation of pro-inflammatory prostaglandins. A prospective, randomized, controlled, double-blind studies are necessary to confirm the findings of this open-label, pilot study.

## Introduction

Oral mucositis is a common toxicity associated with cytotoxic chemotherapy used for cancer treatment and results in severe discomfort and impairs patients’ ability to eat, swallow, and talk. Mucositis also has an indirect effect on tumor outcomes as its presence often necessitates an unfavorable modification of anti-cancer therapy such as breaks in chemotherapy or a dose reduction of chemotherapy [1-3].

Mucositis risk varies among patients with colorectal cancers who receive multicycle chemotherapy. Although the reported cycle 1 incidence varies, about 15% - 20% of patients being treated with commonly used chemotherapy regimens for cancers reportedly develop ulcerative oral mucositis [3, 4].

One of the factors associated with chemotherapy-induced oral mucositis (COM) exacerbation is the activation of cyclooxygenase pathway that mediates ulcer and pain through the upregulation of pro-inflammatory prostaglandins [5]. Chemotherapy-induced myelosuppression places patients at significant risk of bacteremia and sepsis from oral microorganisms resulting in increased COM [1-3, 6].

Hangeshashinto (TJ-14), a Japanese traditional medicine (kampo) [7], has been reported to downregulate the pro-inflammatory prostaglandins, such as prostaglandin E2 in colitis animal model [8, 9]. Moreover, one of the main ingredients of TJ-14, berberine, with broad-spectrum antibacterial activity has been shown to inhibit butyrate-induced colonic epithelial cell death [10, 11].

In light of the purported anti-inflammatory and antibacterial activities of TJ-14 in experimental models, we investigated whether TJ-14 had beneficial effects on COM in patients with advanced colorectal cancer.

## Patients and Methods

We enrolled 14 patients with advanced colorectal cancer who underwent chemotherapy at Asahikawa Medical University Hospital and Higashi-Asahikawa Hospital from January 2009 through August 2010. Details of the study and testing procedures were explained, and a written informed consent was obtained from each participant. Fourteen patients who agreed to participate in the study by signing the written informed consent. All study participants were afebrile patients with lesions mostly on the movable mucosa of the buccal mucosa and lateral and ventral surfaces of the tongue that appeared 7 to 10 days after the chemotherapy. Table 1 summarizes the patient characteristics. The present study was conducted in accordance with the guidelines for the care for human study adopted by the ethics committee of Asahikawa Medical University and Higashi-Asahikawa Hospital.

**Table 1:** Patient Characteristics

		No. of Patients (%)	
Gender	male	6	(43)
	female	8	(57)
Age	mean	62	
	range	34-80	
PS	0	12	(86)
	1	2	(14)
	2	0	
Concurrent Chemotherpy			
	FOLFOX	5	(36)
	FOLFIRI	9	(64)
Neutropenia			
	Yes	12	(86)
	No	2	(14)
Oral mucositis			
	G1	2	(14)
	G2	6	(43)
	G3	5	(36)
	G4	1	(7)

### Grading of oral mucositis

The severity of COM was graded by two blinded physicians using the Common Terminology Criteria for Adverse Events v4.0 (CTCAE). For the characterization of mucositis, CTCAE v4.0 grades were defined as follows: 1) grade 0: no mucositis; 2) grade 1: asymptomatic or mild symptoms; 3) grade 2: moderate pain, does not interfere with oral intake but modified diet is indicated; 4) grade 3: severe pain, interferes with oral intake; 5) grade 4: life-threatening consequence requiring urgent intervention.

### TJ-14 application

The patients prepared a total 50 ml oral rinse solution with 2.5 g of TJ-14 (Tsumura & Co., Tokyo) and tap water and rinsed their oral mucosa three times daily after each meal. Patients were instructed to hold the solution in the mouth for 10 seconds and then expectorate it. Additionally, TJ-14 was topically applied with a cotton pellet on the oral mucositis at the time of ulcer lesion presentation. Food and drinks were prohibited within 30 minutes of each mouthwash. Treatment continued daily for 7 days. None of the patients received cryotherapy or other mucosal treatment concurrently with chemotherapy. No other mucosal treatment was used with over-the-counter drugs or other medications during the study.

### Statistical analysis

All values were expressed as mean ± standard deviation of the mean (SD). Statistical calculations and analyses were performed with the use of Prism 5 (GraphPad Software, Inc., San Diego, CA) statistical software package for comparing the grades of mucositis before and after TJ-14 treatment. Mann-Whitney test was used. All statistical tests performed were two-sided. Differences were considered to be significant at *P* < 0.05.

## Results

**Figure 1:**
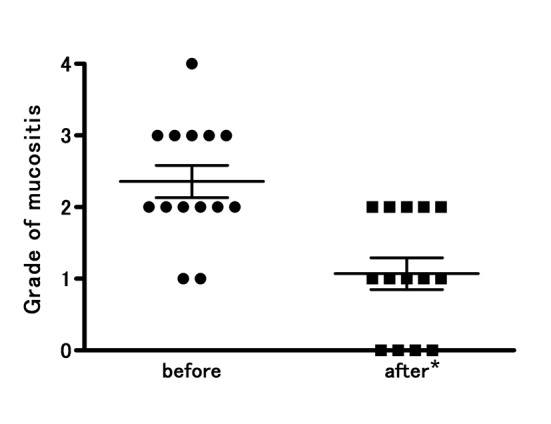
Improved CTCAE grades for oral mucositis following topical treatment of the oral mucosa with hangeshashinto (TJ-14). There was significant reduction in all the grades mucositis from 2.4 ± 0.8 to 1.1 ± 0.8 (* p = 0.0012). The severity of chemotherapy-induced oral mucositis was graded according to the Common Terminology Criteria for Adverse Events v4.0. Results are expressed as mean ± SD.

Fourteen patients with COM received TJ-14 and completed the study. The compliance was good and side effects from TJ-14 were not observed during the study period. Prior to TJ-14 treatment, one patient had grade 4, five patients had grade 3, six patients had grade 2, and two patients had grade 1. After the one-week TJ-14 topical treatment, the patient with grade 4 mucositis improved to grade 2. Of the five patients with grade 3 mucositis, three improved to grade 2 and two improved to grade 1. Among the six patients with grade 2 mucositis, three improved to grade 1, two improved to grade 0, and one had no change. Two patients with grade 1 mucositis improved to grade 0. None of the patients became worse compared to baseline. At the end of the study, thirteen of the fourteen patients (92.8 %) showed improvements in oral mucositis, with significantly decreased mean CTCAE grades (*P* = 0.0012) (Fig. 1).

## Discussion

We found that repeated topical application of TJ-14, even on a short-term basis, improved the severity of COM symptoms in the majority of patients. We also noted that two-thirds of patients on FOLFIRI treatment developed COM.

The current standard of care for patients with advanced colorectal cancer includes varied schedules of chemotherapy like FOLFOX (leucovorin, 5-FU, and oxaliplatin) or FOLFIRI (leucovorin, 5FU, and irinotecan), with FOLFIRI posing a slightly higher risk of developing COM (35%) [4]. Mucosal lesions typically appear between 7 and 14 days after the initiation of chemotherapy [6, 12], mainly on the movable mucosa and rarely affecting the dorsum of the tongue, the hard palate, or the gingiva [3]. In this study, we noted similar observations in our patients, notably a higher incidence of COM among those on FOLFIRI regimen.

Previous reports implicate that some of the exacerbating factors of COM include pro-inflammatory cytokines, nitric oxide, ceramide, matrix metalloproteinases, and pro-inflammatory prostaglandins that lead to apoptosis and tissue injury [1, 3, 5]. Moreover, because prostaglandin E2, a pro-inflammatory prostaglandin, is known to act at pain receptors on neurons and to mediate tissue injury via release of matrix metalloproteinase [5, 13], targeted therapy to downregulate the cyclooxygenase pathway and inhibit prostaglandin synthesis appears important for pain control and mucosal healing in COM [5]. TJ-14 has been reported the anti-inflammatory effects, such as inhibition of pro-inflammatory prostaglandins, including prostaglandin E2, cytoplasmic phospholipase(cPLA), and COX-2 in both in vivo and vitro studies [8, 9]. In clinic, TJ-14 has been used for inflammatory diseases such as gastrointestinal catarrh and gastritis [14]. Although the precise mechanisms remain elusive, TJ-14 might show the anti-COM effect via downregulation of pro-inflammatory prostaglandins, especially prostaglandin E2.

As most of the patients in the study had neutropenia, we also suspected the dynamic alteration of oral microbiota in promoting infective processes which occur more readily in the presence of neutropenia (as in chemotherapy patients) or other deterioration in host defense systems that causes reduced levels of salivary IgG, IgA, and IgM [15]. Myelosuppression is known to increase gram-negative organisms. Bacterial cell wall products such as lipopolysaccharide (LPS) amplify mechanisms that exaggerate and extend the injury by stimulating infiltrating macrophages to produce additional damaging cytokines [3, 6, 12].

TJ-14 is comprised of 7 extracted components: Pinelliae Tuber, Scutellariae Radix, Zingiberis Rhizoma, Ginseng Radix, Glycyrrhizae Radix, Zizyphi Fructus, and Coptidis Rhizoma. Berberine is the main ingredient of Coptidis Rhizoma with strong and wide-spectrum antimicrobial activity [10, 11]. We therefore speculated that compounds like berberine might be responsible for the antimicrobial and anti-inflammatory effects, suppression of prostaglandins, and alleviation of COM symptoms induced by TJ-14.

In conclusion, our findings support the possible benefits of TJ-14. This must be counter-balanced by cautions that further controlled, double blind studies are needed to confirm the findings.
